# Classification of voluntary cough sound and airflow patterns for detecting abnormal pulmonary function

**DOI:** 10.1186/1745-9974-5-8

**Published:** 2009-11-20

**Authors:** Ayman A Abaza, Jeremy B Day, Jeffrey S Reynolds, Ahmed M Mahmoud, W Travis Goldsmith, Walter G McKinney, E Lee Petsonk, David G Frazer

**Affiliations:** 1 National Institute for Occupational Safety and Health, Health Effects Laboratory Division, Pathology and Physiology Research Branch, 1095 Willowdale Road, Morgantown, West Virginia, USA; 2 Department of Computer Science and Electrical Engineering, West Virginia University, Morgantown, West Virginia, USA; 3 Department of Mechanical and Aerospace Engineering, West Virginia University, Morgantown, West Virginia, USA; 4 Department of Medicine, West Virginia University School of Medicine, Morgantown, West Virginia, USA

## Abstract

**Background:**

Involuntary cough is a classic symptom of many respiratory diseases. The act of coughing serves a variety of functions such as clearing the airways in response to respiratory irritants or aspiration of foreign materials. It has been pointed out that a cough results in substantial stresses on the body which makes voluntary cough a useful tool in physical diagnosis.

**Methods:**

In the present study, fifty-two normal subjects and sixty subjects with either obstructive or restrictive lung disorders were asked to perform three individual voluntary coughs. The objective of the study was to evaluate if the airflow and sound characteristics of a voluntary cough could be used to distinguish between normal subjects and subjects with lung disease. This was done by extracting a variety of features from both the cough airflow and acoustic characteristics and then using a classifier that applied a reconstruction algorithm based on principal component analysis.

**Results:**

Results showed that the proposed method for analyzing voluntary coughs was capable of achieving an overall classification performance of 94% and 97% for identifying abnormal lung physiology in female and male subjects, respectively. An ROC analysis showed that the sensitivity and specificity of the cough parameter analysis methods were equal at 98% and 98% respectively, for the same groups of subjects.

**Conclusion:**

A novel system for classifying coughs has been developed. This automated classification system is capable of accurately detecting abnormal lung function based on the combination of the airflow and acoustic properties of voluntary cough.

## Background

Cough is a natural respiratory defense mechanism to protect the respiratory tract and one of the most common symptoms of pulmonary disease [1]. There is a growing interest in using the characteristics of voluntary cough to detect and characterize lung disease [2,3]. Currently, no standard method for automatically evaluating coughs has been established, even though a variety of approaches have been reported in the literature [4,5].

A cough is normally initiated with an inspiration of a variable volume of air, followed by closure of the glottis, and contraction of the expiratory muscles that compresses the gas in the lungs. These events occur immediately before the sudden reopening of the glottis and rapid expulsion of air from the lungs. When flow limitation is reached during coughs that begin at the same lung volume, the airflow and acoustic properties are repeatable and unique for a given subject [6].

There are many examples in the literature that describe methods to analyze cough characteristics based on the subjective interpretation of cough sound recordings and the analysis of spectrograms [4,5,7-12]. In those studies, the acoustical signals were normally recorded either at the neck, over the trachea, or on the chest wall using a contact microphone while the respiratory phase was recorded simultaneously by measuring the airflow from the mouth. In one case, Murata *et al*. [8] described the ability to discriminate acoustically between productive and non-productive cough by the analysis of time expanded waveforms combined with spectrograms. In another instance, Van Hirtum *et al. *[13], were among the first to describe an automated classifier that could differentiate between 'spontaneous' and 'voluntary' human coughs generated by a given individual. They recorded free field cough sounds and were able to identify several distinguishing features of the acoustic signals. Neural networks and fuzzy classification methods were used to make a distinction between coughs in a database that included 12 individual subjects.

The aim of the present study was to develop a new method to characterize and classify the acoustical and airflow properties of human voluntary coughs based on previous work [14]. Cough airflow and acoustic properties of voluntary coughs from subjects with normal and abnormal lung function were recorded using a high fidelity system that has been described previously [14]. A low computational-cost classification system was then developed and evaluated on its ability to identify individuals with respiratory disorders based entirely on a feature set extracted from the recorded cough airflow and acoustic signals. Feature redundancy and extraneous noise were minimized using a principal component analysis. These features were used by an eigenvector classification technique to identify differences in cough characteristics between populations of test subjects. The classification technique was evaluated by comparing the results of the cough analysis with the diagnosis of pulmonologists.

## Methods

### Cough Recording System

**Figure 1:**
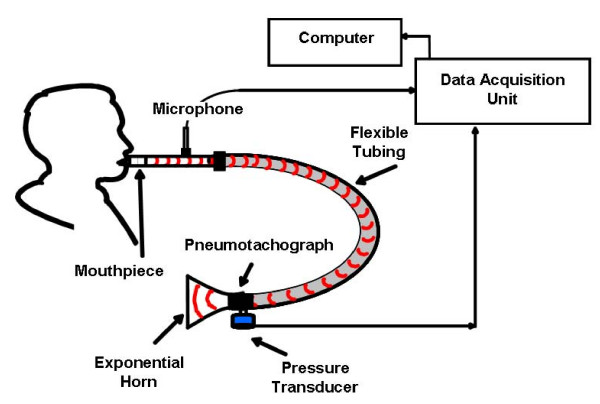
**The high fidelity system used to simultaneously record sound pressure waves and airflow during a cough**.

A block diagram of the system that was designed to record high fidelity cough sound and airflow measurements is illustrated in Figure 1. The system was composed of a cylindrical mouthpiece attached to a 1" diameter metal tube with a 1/4" microphone (Model 4136, Bruel & Kjaer, Norcross, GA) mounted at a 90° angle with its diaphragm tangent to the metal tube. A 1" diameter, 13' long, gum rubber flexible tube was attached to the metal tube opposite the mouthpiece. A pneumotachograph (Model 2, Fleisch, Lausanne, Switzerland) and differential pressure transducer (Model 239, Setra systems, Boxborough, Maryland) were employed at the terminal end of the flexible tube to measure airflow during a cough. The system was terminated with an exponential horn to reduce acoustic reflections. The calibration and accuracy of the system have been discussed previously [14].

A software "virtual instrument" was designed using LabVIEW to capture the sound pressure and flow signals generated as a subject coughed through the mouthpiece. The virtual instrument allowed the user to select the sampling frequency, total sampling time, high-pass filter characteristics, input signal range, and triggering considerations. Under normal operation, a high-pass filter with a cut-off frequency of 22.4 Hz, and an anti-aliasing filter with a cut-off frequency of 25.6 kHz were applied to the signal. The frequency response of the condenser microphone was 20 Hz to 35 kHz (± 1 dB). This system was capable of performing spectral analysis of cough sound signals in the frequency domain between 50 Hz and 25 kHz.

**Figure 2:**
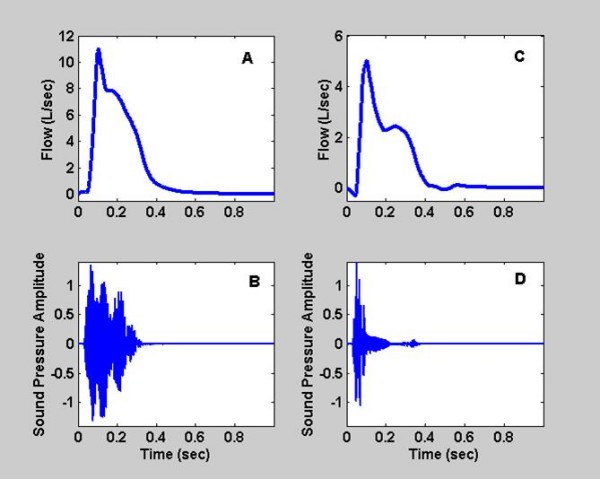
**Airflow and sound pressure wave measured during a voluntary cough**. A and B display the signals for a normal subject. C and D show the corresponding measurements for a subject with abnormal lung physiology.

**Figure 3:**
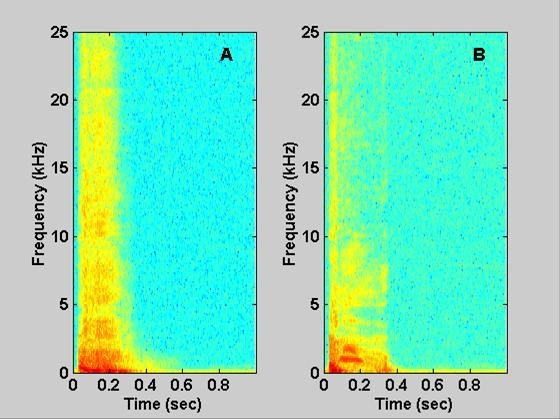
**Spectrograms of sound signals for voluntary coughs**. A shows the joint time-frequency relationship from the normal cough shown in Figure 2A. B shows the relationship from the abnormal cough shown in Figure 2C. Note: the highest intensity is represented by red then yellow and is dark blue at its lowest values.

Figure 2 shows examples of cough sound pressure waves and airflow measurements for coughs from a normal subject and a subject with abnormal lung function. Spectrograms of these cough sound signals are displayed in Figure 3.

### Cough Data Collection

The testing procedure was approved by the Institutional Review Board of West Virginia University and standardized using the following protocol. Subjects first viewed a short video describing the correct performance of a voluntary cough. This was to ensure that all coughs from a particular subject were repeatable. Test subjects were coached to keep their glottis open to prevent sound generated due to the glottis closing at the end of the cough. Before beginning a cough, each individual was asked to inhale to total lung capacity (TLC), relax and exhale. This was followed by a second inhalation to TLC at which time the subject was asked to form a seal with their teeth and lips around the mouthpiece connected to a metal tube (as shown in Figure 1), and to cough vigorously. Three successive individual coughs were recorded to ensure that they had a repeatable flow-volume relationship.

A total of 58 male and 54 female subjects were tested. There were 27 male and 25 female subjects classified as normal, as well as 31 male and 29 female subjects classified as having abnormal lung function. All test subjects were examined at the pulmonary function laboratory of Ruby Memorial Hospital, after providing informed consent. The study protocol was reviewed and approved by the local institutional review board, and all participants gave written informed consent. The diagnosis of a pulmonary disease was based upon a pulmonary physician's review of all the available information pertaining to each patient. This included the course of symptoms, findings reported on the physical examination, medical records, pulmonary function tests, and other laboratory results including radiographic images. In addition, risk factors reported under personal, social, occupational and family history were considered. The pulmonary function tests were performed using a whole body plethysmograph (Model 1085/D, MedGraphics, St. Paul, Minnesota) and spirometer (Model Jaeger MasterScope, VIASYS Healthcare, Hoechberg, Germany). Those subjects who were diagnosed with either restrictive or obstructive lung disorders were considered to have abnormal lung function. Those subjects that the pulmonologist diagnosed as disease-free were considered to be normal. Test subject population demographics, including pulmonary function test indices, are shown in Table 1.

**Table 1:** Description of group populations of test subjects.

	Normal Male (n = 27)*	Lung Disease Male (n = 31)**	Normal Female (n = 25)***	Lung Disease Female (n = 29)**
Age (years)	51.19 ± 16.71	58.48 ± 9.88	52.12 ± 16.73	56.31 ± 14.53

Height (cm)	177 ± 10	173 ± 7.0	160 ± 7.0	160 ± 7.0

Weight (kg)	93.30 ± 20.02	88.48 ± 30.16	83.29 ± 27.13	76.8 ± 22.52

**Smoking History**				

Never	9	3	13	8

Former	15	19	9	14

Current	3	9	3	7

**FEV1 % Predicted**				

(>79) %	27	1	24	4

(60-79) %	0	15	0	13

(40-59) %	0	12	0	8

(<40) %	0	2	0	3

**FVC % Predicted**				

(>79) %	26	16	23	11

(60-79) %	0	12	0	9

(40-59) %	0	2	0	6

(<40) %	0	0	0	2

**FEV1/FVC % Predicted**				

(>88) %	23	9	23	14

(70-88) %	3	6	0	10

(60-69) %	0	8	0	0

(40-59) %	0	6	0	3

(<40) %	0	1	0	1

* One subject in this group was evaluated without a FVC measurement.

** One subject in each group of these two groups was diagnosed without spirometry.

*** One subject in this group was evaluated without a FVC measurement and one was evaluated without spirometry measurements.

### Feature Extraction

Cough sound and airflow signals were analyzed in both the time and frequency domains and representative features were extracted from both signals. There were 29 features based on time (5 were sound-based, and 24 were airflow-based), and 108 features based on frequency (106 were sound-based, and 2 were airflow-based). These features are described in detail in Tables 2 and 3. The extracted features were normalized with respect to their maximum value and had a range between 0 and 1.

**Table 2:** Cough flow signal extracted features.

	*Time Series*
1	Peak cough flow (L/s)

2	Average cough flow (L/s)

3	Maximum cough flow acceleration(L/s^2^)

4	Total cough volume (L)

5	Time at which 25% cough volume has been expelled/time at which 100% cough volume has been expelled

6	Time at which 50% cough volume has been expelled/time at which 100% cough volume has been expelled

7	Time at which 75% cough volume has been expelled/time at which 100% cough volume has been expelled

8	25% total time of cough/cough volume

9	50% total time of cough/cough volume

10	75% total time of cough/cough volume

11	Time at peak flow/total time

12	Crest Factor: maximum flow/Root Mean Square "RMS" flow

13	Form Factor: RMS flow/mean flow

14	Transit time: (s)

15	Skewness: where μ, and σ are the mean, and the standard deviation of the cough airflow signal respectively.

16	Kurtosis: where μ, and σ are the mean, and the standard deviation of the cough airflow signal respectively.

17	Cough flow variance

18	Cough flow variance normalized with respect to volume

19-20	The top two principal components for flow*

21-22	The top two principal components for volume*

23-24	The top two principal components for Acceleration*

	***Frequency Series***

25	Beta: the inverse power law 1/f^β ^of the power spectrum [22].

26	Wavelet parameter based on the variability in the wavelet detail coefficients found in the wavelet decomposition of the cough flow

*Only the first two principal components were used, as experimentally the accuracy started to drop afterwards.

**Table 3:** Cough sound signal extracted features.

	*Time Series*
1	Cough Length: length from the start of the cough until 99.4% of the cough energy is achieved (s)

2	L-ratio: Cough flow length/cough sound length

3	Skewness: where μ, and σ are the mean, and the standard deviation of the cough sound signal respectively.

4	Kurtosis: where μ, and σ are the mean, and the standard deviation of the cough sound signal respectively.

5	Crest Factor: maximum sound pressure wave/Root Mean Square "RMS" sound

	***Frequency Series***

6	Dominant Frequency: the frequency with the most power present in the cough sound pressure wave (Hz)

7	Total energy

8-24	Octave Analysis (1-17)**

25	Total Power: total power in the cough sound signal (W)

26	Peak Power: maximum power level (W)

27	Average Power: Average power over all frequency ranges (W)

28	Sound beta: the inverse power law 1/f^β ^of the power spectrum [22].

29	Sound Wavelet: a wavelet parameter based on the variability in the wavelet detail coefficients found in the wavelet decomposition of the cough sound

30	Ratio: mean spectrogram intensity/max spectrogram intensity

31	Peaks: this counts the number of peaks in the spectrogram that meet a given threshold

32-51	Spec1 - Spec20: The spectrogram is broken into 20 evenly spaced time intervals. For each interval, the maximum energy is found, and the corresponding frequency is saved.

52-81	Spec21 - Spec50: The spectrogram is broken into 30 evenly space time intervals. For each interval, the average frequency is calculated and saved.

82-111	Spec51 - Spec80: The spectrogram is broken into 30 evenly spaced frequency intervals. For each frequency interval the time at which half of the energy is attained is saved.

**Octave analysis: the power of cough sound pressure wave is broken into octaves (frequency bands) and the power found in each octave is calculated in each band. Analysis was stopped at 18,102 Hz, because only 2% of the energy remains above Oct17.

### Classification Method

The classification system presented in this study was based on the establishment of subspaces corresponding to each cough class using the principal components of the training samples from each class. The projections of the unclassified cough features onto these subspaces formed the foundation of the classification technique. Since there is some resemblance between this method for cough classification and the eigenfaces method [15], the resulting basis vectors defining the cough feature subspaces have been described as eigencoughs. A principal component analysis of the features extracted from the cough airflow and sound signals was used to construct the class subspaces. The training coughs for each class were selected. For each set of training samples, construction of the subspaces proceeded as follows.

The average of the class ('C_1_', 'C_2_'...'C_M_') samples is computed as(1)

where *N*_*ω *_is the number of exemplars of class *ω*, and *x*_*iω *_is the feature vector of the i^th ^exemplar of class *ω*. Now let(2)

represent the matrix of the average-adjusted sample of class *ω*. Next, the eigenvectors *u*_*iw *_of the scatter matrices of each class sample were computed using the efficient technique proposed in [15], by first solving the eigenvalue problem:(3)

where *λ*_*jω *_was the *j*^*th *^eigenvalue, and *v*_*jω *_is the *j*^*th *^eigenvector of matrix (). Finally, *v*_*jω *_was linearly mapped to *u*_*jw *_using:(4)

**Figure 4:**
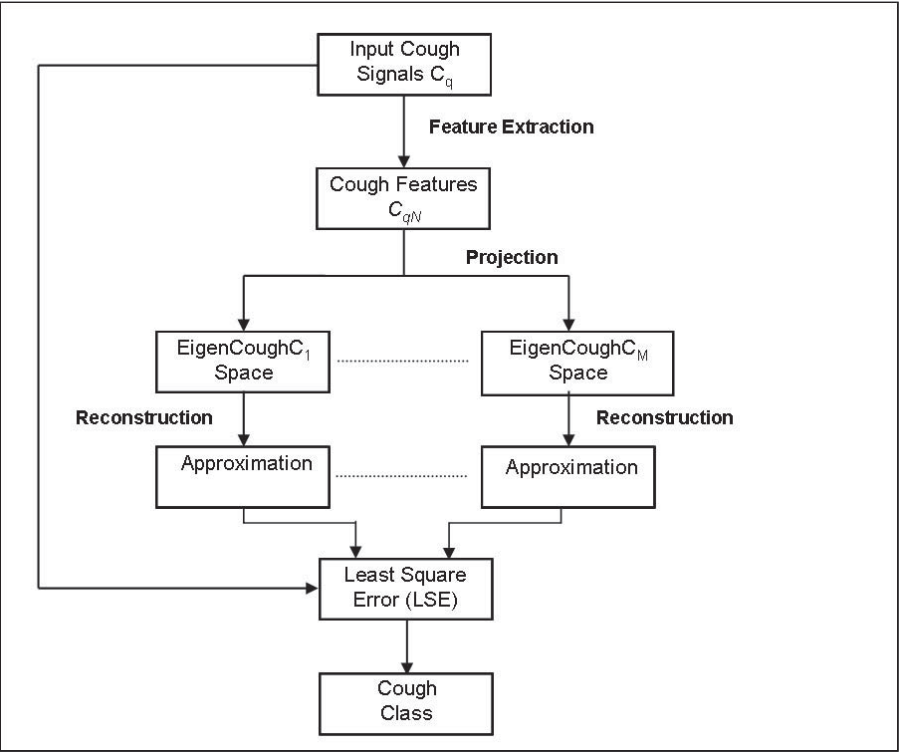
**Cough reconstruction and classification method**.

The eigenvectors were then arranged in a descending order based on their corresponding eigenvalues. To differentiate between normal and diseased cough, only the first K eigenvectors were selected for the subspace projection. Values of K were tested based on either the preservation of 95% of the energy or a reduced number of eigenvectors as described in [15,16]. The final value of K that produced the most accurate classification results was chosen. Once the vector subspaces were constructed, individual coughs were classified as illustrated in Figure 4. First the set of features of an unclassified (novel) cough (C_q_) were extracted and normalized (*C*_*qN*_). Then values of (*C*_*qN*_) were projected onto each of the cough class subspaces to obtain the following set of weight coefficients as described by equation (5):(5)

In the above expression *μ*_*ω *_represents the mean vector, and *u*_*jω *_is the j^th ^eigenvector of class *ω*. The weight sets were then used along with the sample means to reconstruct *C*_*qN *_in each class subspace, thus obtaining the approximations :(6)

Next the representation error between *C*_*qN *_and its approximation in each class was determined as follows:(7)

Finally, the novel cough coefficient *C*_*q *_was assigned to class *ω *based on the least square error rule as follows:(8)

To assess the sensitivity and specificity of the classification system, the Receiver Operating Characteristic (ROC) curve [17] was constructed using the following assignment rule:(9)

where *r *ranges from minimum to maximum values of the ratio . The sensitivity and specificity of the classification method are found as follows:

The overall performance or discriminative rate was defined as:

### Experimental Design

The dataset used in this research consisted of three coughs each from 58 male subjects (31 diseased, 27 normal) and 54 female subjects (29 diseased, 25 normal). Male and female training sets were considered separately. All the coughs from each of the test subjects were used to train the classifier with the exception of the three coughs from one subject [17]. The three withheld coughs were then analyzed individually. If at least two out of the three coughs were classified as either normal or abnormal, the subject was assumed to be a member of that group. This procedure was repeated until every subject had been evaluated.

## Results

### Results of Pulmonary Function Measurements

The results of lung function measurements made in the pulmonary laboratory at Ruby Memorial Hospital, West Virginia University, are shown in Table 1. The average value (± SD) for the age, height, and weight of each group of test subjects are also given along with their smoking history. Pulmonary physicians' diagnoses were used to determine if subjects had normal or abnormal lung function. Table 1 also indicates the number of subjects within percent predicted ranges of their FEV_1.0_, FVC, and FEV_1.0_/FVC ratio. Most test subjects with abnormal lung function had mild to moderate impairment. Three voluntary coughs from each of these subjects were analyzed to determine if their cough airflow and acoustic characteristics could be used to establish if they had normal or abnormal lung function.

### Results of Classifying Voluntary Coughs

**Figure 5:**
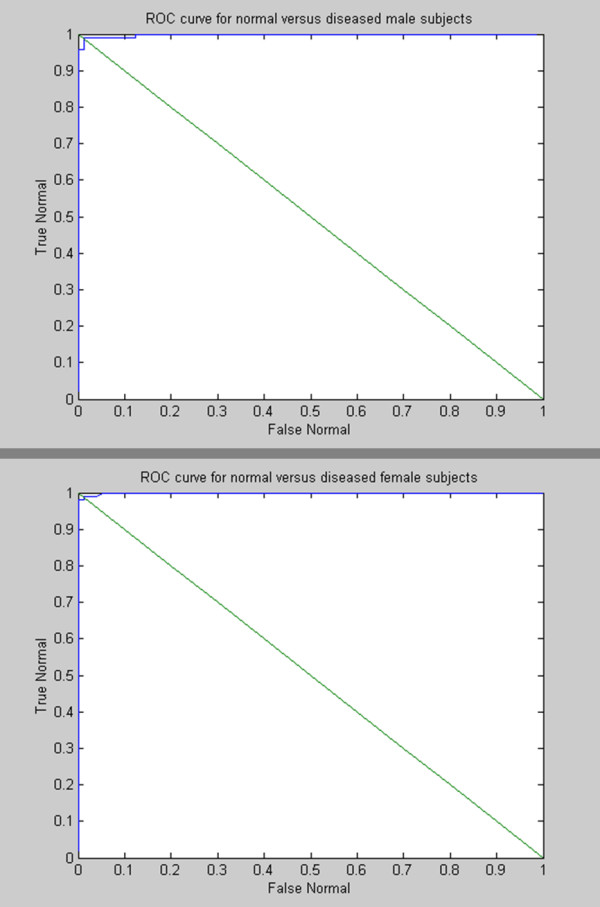
**ROC curves of classification results for normal versus diseased coughs of male and female subjects**.

The results of the eigencough method for distinguishing between coughs of normal subjects and subjects with lung disease are shown in Table 4. The overall performance of our optimal classifier was 94% for coughs from female subjects and 97% for coughs from male subjects (K was chosen to preserve 95% of the total energy). The ROC curves for coughs from each gender are shown in Figure 5. The point on the curve which yielded an equal sensitivity and specificity was 98% for coughs from female subjects and 98% for coughs from male subjects, respectively. Several preliminarily experiments were performed to test and adjust the parameters of the classification method to improve its ability to discriminate between coughs of normal subjects and those with lung disease. Comparisons were made between the results using only the cough airflow features, the cough sound features, or the fused features from both signals [18]. When the fused features were used, the overall classification accuracy reached 94% and 97% for coughs from female and male subjects respectively. This was compared to accuracies of 85% and 91% for flow features only and 93% and 91% for sound features only (K was chosen to preserve 95% of the total energy).

**Table 4:** Classification accuracy for normal versus diseased coughs.

		System Output for Male Coughs	
		**Diseased** **(Obst. & Rest.)**	**Normal**

True Class	Diseased (Obst. & Rest.)	94%	6%
	
	Normal	0%	100%

Overall Performance 97%			

		**System Output for Female coughs**	

		**Diseased** **(Obst. & Rest.)**	**Normal**

True Class	Diseased (Obst. & Rest.)	90%	10%
	
	Normal	0%	100%

Overall Performance 94%			

A second experiment was performed to determine the optimum number of principal components (K) used by the classifier. According to the literature [15,16], K has usually been selected to preserve either 90%, 95%, or 99% of the total energy. It was determined that the overall classification accuracy in this study was 94% and 97% when K was chosen to preserve 95% of the total energy for female/male subjects. This can be compared to 94% and 93% for the case in which K preserved 90% of the energy and 91% and 95% when K preserved 99% of the energy. This indicated that some features may have introduced noise which reduced the accuracy of the classifier.

## Discussion

The goal of this study was to determine if the characteristics of voluntary coughs could be used to distinguish between individuals with normal and abnormal lung function. The approach was to measure a wide variety of features describing both the acoustical and airflow characteristics of a voluntary cough in both the time and frequency domains. It should be pointed out that the features were selected arbitrarily and there was no attempt to optimize their selection. Once they were determined, all the features were normalized with respect to their maximum values. The next step was to use a principal component analysis to eliminate redundant information contained in the feature set. Then, the principal components of the features were used to define a reduced number of orthogonal vectors representing each cough.

A unique approach for developing a classifier for categorizing voluntary coughs was used that was based on the subspace projection of the principal components into a vector space. One of the most important parameters of the classifier was determining K, the number of principal components needed in the analysis. The initial expectations were that the results would be more accurate using the highest value of K. This was not the case, however, and inclusion of some of the cough parameters appeared to increase noise. It was found in preliminary experiments that increasing K to preserve 95% of the energy contained in the data sets enhanced the performance of the classifier. In contrast, however, for both female and male groups, the classifier performance deteriorated when K was increased to preserve 99% of the energy in the cough parameters.

Due to the limited number of samples, the classifier was trained using all the data from all the subjects in each group except one. The coughs of that subject were evaluated using the trained system. This process was repeated for each member of the male and female test groups.

An analysis of the overall performance of our optimal classification system showed that there were 3 misclassifications within the group of the 58 male subjects. There were 0 subjects with normal lung function that were classified as having abnormal lung function and 3 subjects who had abnormal lung function but were identified as having normal lung function. Out of the total population of 54 women subjects, 3 were misclassified. There were 0 subjects with normal lung function who were classified incorrectly and 3 subjects with abnormal lung function who were recognized as having normal lung function. Figure 5 shows the sensitivity and specificity of the cough analysis method for detecting abnormal lung function in male and female test subjects. The classification criteria can be chosen so that a sensitivity and specificity can be selected depending upon the type of errors that are acceptable for a given testing scheme.

Even though the original feature set was reduced by choosing the largest eigenvectors during the classification process, optimization of the selection of the feature set as well as different methods of feature normalization remains an area of research to be explored. It should also be pointed out that only one type of classifier was tested in the present study. It is possible that for a given feature set, other classifiers using neural networks, genetic algorithms, etc., may provide even better results.

Under certain circumstances, using cough airflow and sound analysis to detect abnormal lung function has several advantages compared with conventional pulmonary function testing methods. First, cough analysis may be useful as a screening method to quickly evaluate changes in lung function of a large population of test subjects in a short period of time. Future studies should evaluate the utility of cough analysis in early disease detection. Experience has shown that subjects show little reluctance to performing a voluntary cough for testing purposes. The procedure is performed easily and quickly and requires a minimum of training since test subjects are usually very familiar with a voluntary cough maneuver. Another advantage is that voluntary coughs can be performed by the very young, the physically challenged, and geriatric subjects who may not be able to easily perform conventional pulmonary function tests. It is also possible that cough feature analysis can be useful in tracking the progression or recovery of pulmonary disorders without performing more strenuous flow-volume tests.

In the future voluntary coughs could be used to distinguish between types of pulmonary disorders such as obstructive and restrictive lung diseases. There is some preliminary evidence that voluntary cough characteristics may be related to changes in specific airway resistance in animals [19] which may also hold true for humans. It should be noted that the accuracy of cough feature analysis could still be improved in a variety of ways. For instance, new features may be identified and extracted to provide additional information and increase the accuracy of the classification system. The acoustic and airflow features could be fused at different levels to improve accuracy [20], and existing features that add noise, but contribute little information to the classification system, could be eliminated [21]. Preliminarily experiments have shown that fusion of the data at the feature level [18] improved the performance of the classifier.

A limitation of this study is that variables such as age, body height, body weight and race, which are known to have an effect on forced pulmonary function indices, were not considered when classifying coughs from test subjects. These factors have been shown to be important when calculating percent predicted values of many pulmonary function indices. As additional test results involving voluntary cough analysis become available, consideration of these parameters should lead to an increased ability of the cough analysis system to discriminate between groups of subjects with normal and abnormal lung function.

It is possible that more appropriate features may be extracted from the data and that other features that do not contribute or even reduce the classification accuracy of the system can be eliminated. However, the classification technique presented in this research provides a highly accurate method of distinguishing between subjects with normal and abnormal lung function based on voluntary cough characteristics.

## Conclusion

This paper describes the development and initial assessment of a unique approach for classifying voluntary coughs from normal subjects and subjects with lung disorders using features extracted from the cough sound and airflow signals. The novel classification system was trained to detect differences between the projection of principal components derived from the features of coughs from male and female test subjects with normal and abnormal lung function. The method is accurate, and can be easily and quickly administered. In the future, cough feature analysis could be used to screen large populations of test subjects in a minimum of time. It is also well suited for testing subjects who may not be able to perform conventional pulmonary function tests.

## Competing interests

The findings and conclusions of this report are those of the authors and do not necessarily represent the views of the National Institute for Occupational Safety and Health.

## Authors' contributions

AAA, JSR, WTG and DGF participated in the design of the study, analyzed the data, and drafted the manuscript. ELP, JBD, AMM, and WGM participated in the design of the study and collected the data. All the authors read and approved the final manuscript.
